# Gene Repositioning Is Under Constraints After Evolutionary Conserved Gene Neighborhood Separate

**DOI:** 10.3389/fgene.2019.01030

**Published:** 2019-10-03

**Authors:** Zhiming Dai

**Affiliations:** ^1^School of Data and Computer Science, Sun Yat-Sen University, Guangzhou, China; ^2^Guangdong Province Key Laboratory of Big Data Analysis and Processing, Sun Yat-Sen University, Guangzhou, China

**Keywords:** genomic neighbors, chromatin regulation, gene expression, yeast, bioinformatics

## Abstract

Genes are not randomly distributed on eukaryotic chromosomes. Some neighboring genes show order conservation among species, while some neighboring genes separate during evolution even though their neighborhoods are conserved in some species. Here, I investigated whether after-separation gene repositioning is under natural selection for evolutionary conserved gene neighborhoods compared with nonconserved neighborhoods. After separation, genes with conserved neighborhoods show low-expression divergence between the after-separation species and the before-separation species. After genes separate from their conserved gene neighbors, their after-separation gene neighbors tend to show coexpression and coprotein complex with their before-separation gene neighbors. These results indicate evolutionary constraints on the selection of neighboring genes after evolutionary conserved gene neighborhoods separate.

## Introduction

Gene order in genomes is not random (Hurst et al., 2004). Genes that tend to remain proximal along DNA (synteny property) show strong transcriptional coexpression in bacterial genomes (Junier and Rivoire, 2016). Neighboring gene pairs tend to show higher coexpression than random gene pairs in yeast *Saccharomyces cerevisiae* (Kruglyak and Tang, 2000; Fukuoka et al., 2004; Lercher and Hurst, 2006), nematode *Caenorhabditis elegans* (Lercher et al., 2003), fruit fly *Drosophila melanogaster* (Boutanaev et al., 2002; Spellman and Rubin, 2002; Bailey et al., 2004; Kalmykova et al., 2005), and mammal human (Singer et al., 2005; Li et al., 2006; Semon and Duret, 2006; Ghanbarian and Hurst, 2015). This coexpression spans up to tens of neighboring genes in *S. cerevisiae* and *D. melanogaster* (Lercher and Hurst, 2006; Weber and Hurst, 2011). Essential genes tend to be genomic neighborhoods in *C. elegans*, *S. cerevisiae* and mouse (Kamath et al., 2003; Pal and Hurst, 2003; Hentges et al., 2007). Genes in specific functional gene groups tend to be neighbors within chromosomes of the human genome (Teichmann and Veitia, 2004; Thevenin et al., 2014). Genes encoding subunits of the same complex tend to aggregate on chromosomes in *S. cerevisiae* (Teichmann and Veitia, 2004). Genes from the same metabolic pathway also show clustering in eukaryotic genomes (Lee and Sonnhammer, 2003).

A range of mechanisms have been proposed to be responsible for the nonrandom gene order in genomes. While proximal genes tend to be coexpressed in bacteria by facilitated coexpression mechanisms independently of the action of transcription factor (TF) (Junier and Rivoire, 2016), TF regulation constrains gene order on yeast chromosomes (Janga et al., 2008), as neighboring genes tend to be coregulated by the same TF (Hershberg et al., 2005; Michalak, 2008). Adjacent genes also show similarity in nucleosome occupancy, chromatin remodeling program, and histone modification pattern (Batada et al., 2007; Chen et al., 2010; Deng et al., 2010). These chromatin structures might influence gene expression and, hence, the conservation of gene order.

Coexpression of neighboring genes still persists after their separation (Wang et al., 2011). Moreover, neighboring genes show interchromosomal colocalization after their separation, and these gene pairs tend to be bound by the same TF (Veron et al., 2011; Dai et al., 2014). These results suggest that gene pairs that were genomic neighbors in the evolutionary past, but currently separated, might be still under similar regulatory programs.

**Figure 1:**
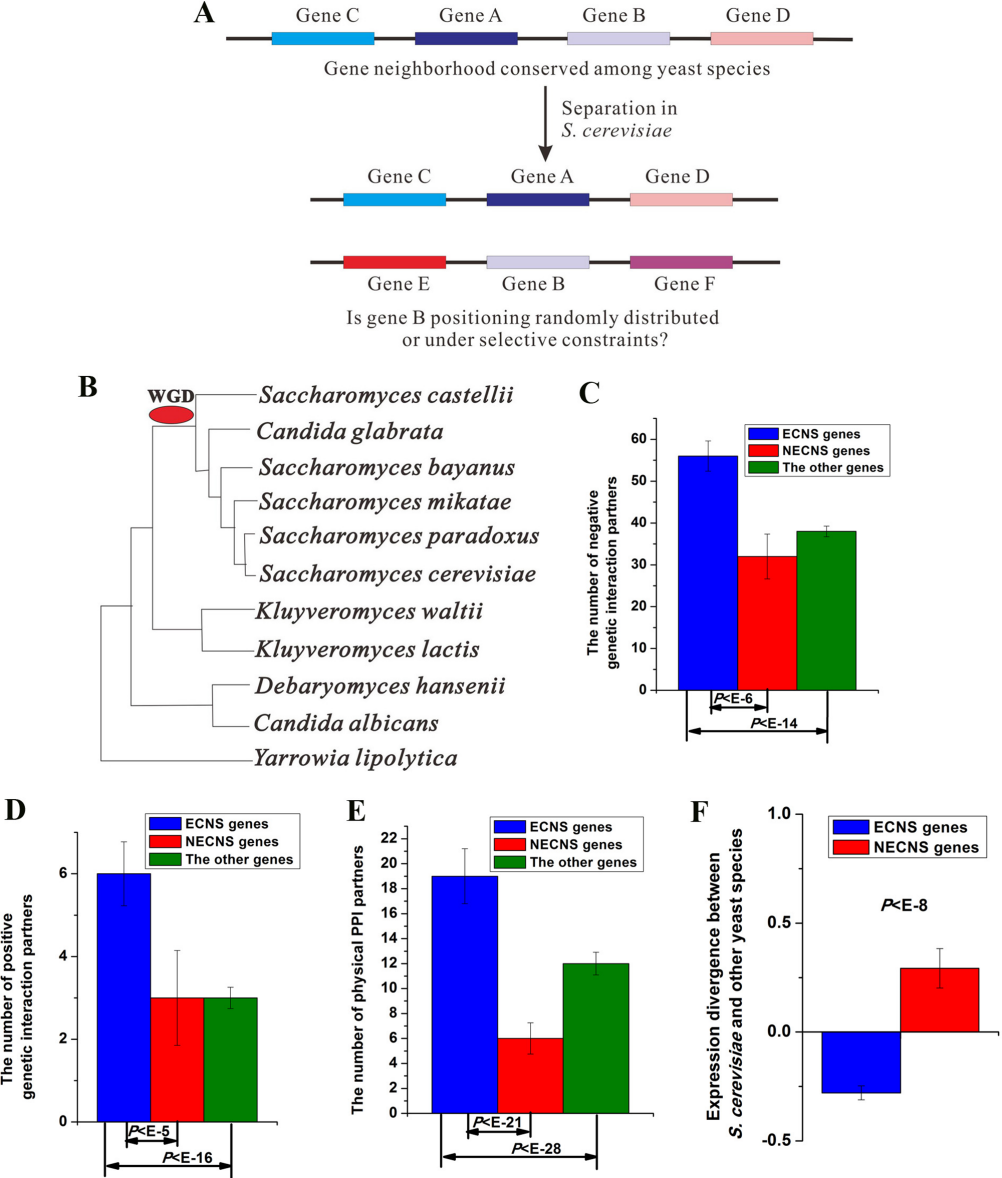
Evolutionary-conserved neighboring genes show low-expression divergence after separation. **(A)** A brief illustration of this study. **(B)** Phylogeny of yeast species included in this study. Median values that correspond to the number of negative genetic interaction partners **(C)**, the number of positive genetic interaction partners **(D)**, the number of physical protein–protein interaction (PPI) partners **(E)**, and expression divergence between *S. cerevisiae* and other yeast species **(F)** were shown for evolutionary-conserved neighbors separated in *S. cerevisiae* (ECNS) and nonevolutionary-conserved neighbors separated in *S. cerevisiae* (NECNS) genes. The statistical significant values calculated from Mann–Whitney *U* test were indicated. Error bars were calculated by bootstrapping.

A considerable number of genes show gene neighborhood conservation over long evolutionary periods (Hurst et al., 2002; Arnone et al., 2012), suggesting that these gene neighborhoods are selectively favorable. If the conservation has functional consequence, conserved gene neighborhoods should maintain links after their separation. Regarding three-dimensional nuclear links, we have previously found that genes showing neighborhood conservation in more species show more interchromosomal colocalization after their separation (Dai et al., 2014). Regarding one-dimensional links, after evolutionary-conserved neighboring genes separate, I asked whether their new positions are under selective constraints (**Figure 1A**). In this study, I focused on yeast species including *S. cerevisiae* to test this possibility since *S. cerevisiae* has been the main model in the studies of gene order, as tremendous amounts of molecular knowledge and data are available.

## Materials and Methods

### Data Preparation

The genome sequences, gene and chromosome annotations of the yeast species, and high-quality predicted gene orthology map across yeast species were obtained from a previous compilation (Wapinski et al., 2007). Gene expression data of yeast species were taken from Thompson et al. (2013). In this study, I used 11 yeast species whose gene annotation data and gene expression data are both available (**Figure 1B**). These include *S. cerevisiae* and its close relatives (*sensu stricto* clade), two other species who have diverged after the whole-genome duplication (*Saccharomyces castellii*, and the human pathogen *Candida glabrata*), two members of the *Kluveroymyces* clade (*Kluyveromyces waltii* and *Kluyveromyces lactis*), two members of the *Candida* clade (the human pathogen *C. albicans* and the halophile *Debaryomyces hansenii*), and *Yarrowia lipolytica*.

Gene expression divergence data in yeast species (including *S. cerevisiae, Saccharomyces paradoxus, Saccharomyces mikatae, and Saccharomyces kudriavzevii*) were taken from Tirosh et al. (2006). The measure of gene expression divergence was based on the interspecies correlations in the gene expression patterns. For each gene, the expression divergence between each two species was corrected by subtracting its value with the average intraspecies divergence of the two corresponding species. The corrected expression divergence for each pairwise species comparison was normalized by subtracting its mean and dividing by its standard deviation, and the normalized values were then averaged over the all pairwise species comparisons; thus, a single value of expression divergence was given for each gene (Tirosh et al., 2006). Genome-wide binding data corresponding to 203 TFs were taken from Harbison et al. (2004). A P value cutoff of 0.005 was used to define the set of genes bound by one particular TF. Protein–protein interaction (PPI) data were taken from Stark et al. (2006). Genetic interaction data were taken from Costanzo et al. (2016). The member genes of cellular component gene sets from S. cerevisiae were downloaded from the Gene Ontology repository (Harris et al., 2004). P values for Gene Ontology process terms were derived using “GOTermFinder” (https://go.princeton.edu/cgi-bin/GOTermFinder). The list of essential genes was taken from Giaever et al. (2002).

### Identification of Two Gene Groups

For each gene in *S. cerevisiae*, I identified its orthologous counterparts in the other 10 yeast species according to a yeast orthology map (Wapinski et al., 2007). I focused on *S. cerevisiae* genes having orthologous counterparts in at least 60% (i.e., 6–10 species) of the other 10 species, resulting in 4,093 genes. I identified *S. cerevisiae* gene pairs whose orthologous counterparts show neighborhoods in more than half (≥ 50%) of their appeared yeast species, meaning that for gene pairs respectively having orthologous counterparts in 6, 7, 8, 9, and 10 species, their orthologous counterparts are gene neighbors in at least 3, 4, 4, 5, and 5 species. The resulting gene pairs were referred to as evolutionary-conserved neighboring gene pairs. I searched for *S. cerevisiae* gene pairs that met two criteria: 1) They are evolutionary-conserved neighboring gene pairs, and 2) they are either on different chromosomes or flanked by at least 15 genes if on the same chromosome in *S. cerevisiae*, that is, they are separated in *S. cerevisiae*. These criteria resulted in 902 gene pairs, which included 1,367 genes. These gene pairs were referred to as yeast evolutionary-conserved neighboring but *S. cerevisiae* separated gene pairs (**Table S1**). Genes in most (853) of the 902 gene pairs are on different chromosomes in *S. cerevisiae*. Orthologous counterparts of the resulting gene pairs tend to remain neighbors in seven yeast species (**Figure S1**). The numbers in the seven species were similar.

Note that the resulting genes might show evolutionary-neighboring conservation with either *S. cerevisiae* neighboring genes, that is, they might not separate from their other evolutionary-conserved neighbors in *S. cerevisiae*, which might cause confusion. Among the 1,367 genes involved in the identified yeast evolutionary-conserved neighboring but *S. cerevisiae* separated gene pairs, I excluded genes in evolutionary-conserved neighboring gene pairs identified above, resulting in 990 genes, and referred to them as genes with evolutionary-conserved neighbors separated in *S. cerevisiae* (ECNS) (**Table S2**).

To control for the evolutionary-conserved neighborhood, I searched for *S. cerevisiae* gene pairs that met two criteria: (1) Their orthologous counterpart are gene neighbors in only 1 of the 10 yeast species, and (2) they are either on different chromosomes or flanked by at least 15 genes if on the same chromosome in *S. cerevisiae*, that is, they are separated in *S. cerevisiae*. I referred to the resulting gene pairs as yeast nonevolutionary-conserved neighboring but *S. cerevisiae* separated gene pairs. To avoid confusion, I excluded genes in evolutionary-conserved neighboring gene pairs from the resulting genes. The refining genes were referred to as genes with nonevolutionary-conserved neighbors separated in *S. cerevisiae* (NECNS).

### Randomized Experiments

I used randomized experiments to test the statistical significance of frequencies of cobound TF, pairwise genetic interactions, pairwise PPI, and cocellular component for gene pairs between before- and after-separation neighboring genes of ECNS genes. For each gene in all gene pairs, I randomly permutated it with one gene that met two criteria: 1) It was on the same chromosomes as the actual gene because some properties like TF binding show preference for some specific chromosomes, and 2) it was not the same gene as the actual gene. In this way, I generated random genes of which the number was the same as that of genes in actual gene pairs. I replaced the actual genes with their corresponding random genes. In this way, I generated random gene pairs of which the number was the same as that of actual gene pairs. I calculated frequencies of cobound TF, pairwise genetic interactions, pairwise PPI, and cocellular component for these random pairs. If the cobound TF, genetic interactions, PPI, and cocellular component were not features of actual gene pairs, the random gene pairs should show a similar degree of frequencies as actual gene pairs. I repeated the randomized experiment 100,000 times to calculate the frequency of experiments having higher degrees than actual gene pairs and referred to this value as *P* value.

## Results

### Evolutionary-Conserved Neighboring Genes Show Low-Expression Divergence After Separation

I studied 11 yeast species whose gene annotation data and gene expression data are both available. I identified 902 *S. cerevisiae* gene pairs, orthologous counterparts of which were gene neighbors in most of their appeared yeast species, but separated in *S. cerevisiae* (see details in Materials and methods section, **Table S1**). However, some genes in my identified gene pairs show evolutionary-neighboring conservation with their *S. cerevisiae* neighboring genes. I excluded these genes to avoid confusion, resulting in 990 genes, and referred to them as ECNS genes (see details in Materials and methods section, **Table S2**). I also identified *S. cerevisiae* gene pairs, orthologous counterparts of which were gene neighbors in only one yeast species, but separated in *S. cerevisiae*. I referred to the resulting genes as NECNS genes. Both ECNS and NECNS genes separated from neighbors of their orthologous counterparts in *S. cerevisiae*; these two gene groups differ in whether their orthologous counterparts show evolutionary conservation in neighborhoods.

I examined the enrichment of ECNS genes for genetic interaction in *S. cerevisiae*. ECNS genes have more genetic interaction partners than NECNS and the other genes (negative interaction:*P* < 10^–6^ for NECNS genes, *P* < 10^–14^ for the other genes; positive interaction: *P* < 10^–5^ for NECNS genes, *P* < 10^–16^ for the other genes; Mann–Whitney *U* test, **Figure 1C, D**). Moreover, ECNS genes have more PPI partners than NECNS and the other genes (*P* < 10^–21^ for NECNS genes, *P* < 10^–28^ for the other genes, Mann–Whitney *U* test, **Figure 1E**). ECNS genes tend to be essential genes compared with NECNS genes (∼22 vs. ∼7%) (Giaever et al., 2002). However, ∼16% of all *S. cerevisiae* genes are essential genes, indicating that NECNS are relatively depleted in essential genes. ECNS genes tend to be involved in housekeeping processes, including cellular process, metabolic process, and biogenesis as indicated by Gene Ontology, whereas NECNS genes tend to be involved in transport process (**Table S3**).

I examined whether ECNS and NECNS genes show similar expression divergence between *S. cerevisiae* and other yeast species (Tirosh et al., 2006). Interestingly, I found that ECNS genes show lower expression divergence than NECNS genes (*P* < 10^–8^, Mann–Whitney *U* test, **Figure 1F**). Moreover, before-separation neighboring genes of ECNS genes also show lower expression divergence than those of NECNS genes (*P* < 10^–13^, Mann–Whitney *U* test, **Figure S2**). These results are consistent with a previous human finding that grouping human genes together along the genome is associated with reduced expression noise (Kustatscher et al., 2017).

### After-Separation Neighboring Genes of ECNS Genes Show Low-Expression Divergence

**Figure 2:**
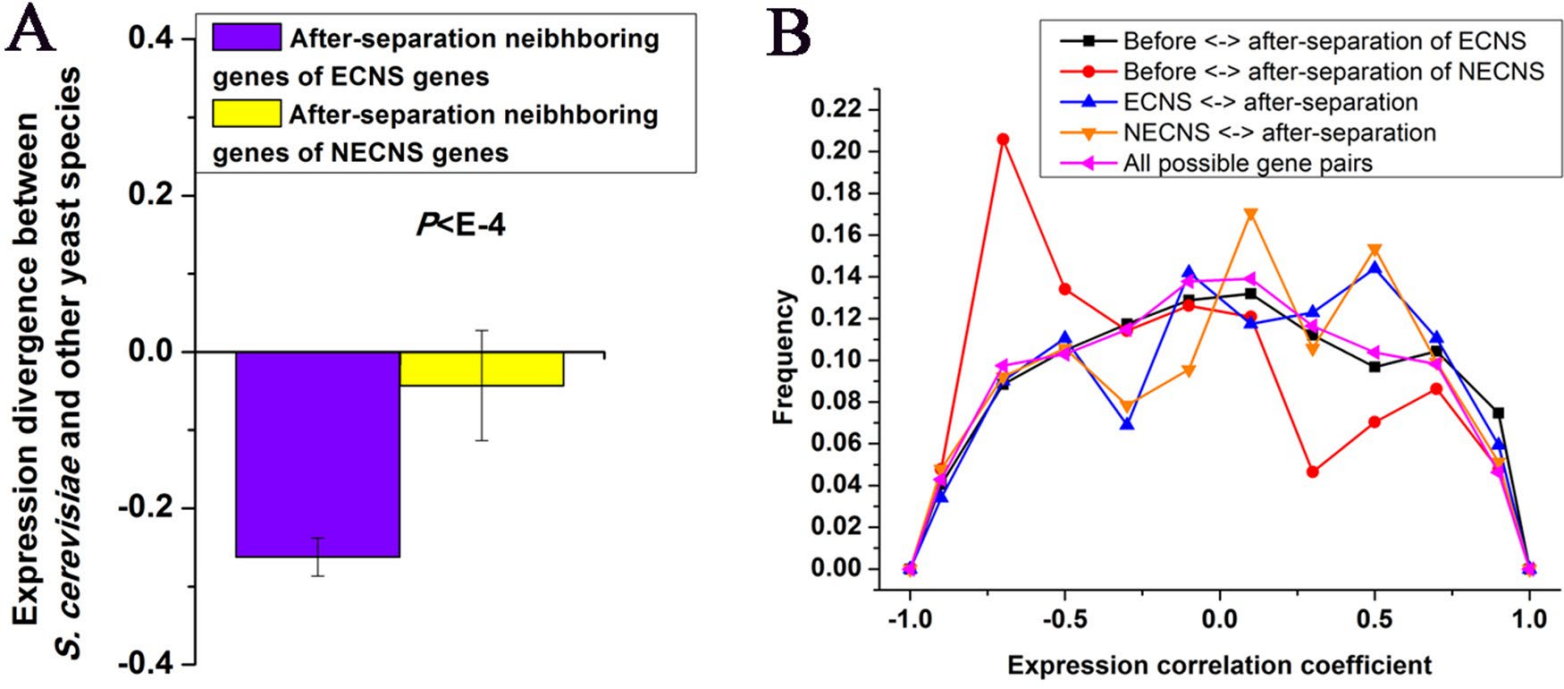
Before- and after-separation neighboring genes of ECNS genes show high coexpression. **(A)** Median values that correspond to expression divergence between *S. cerevisiae* and other yeast species were shown for after-separation neighboring genes of either ECNS genes or NECNS genes. Error bars were calculated by bootstrapping. **(B)** For each gene pair between before- and after-separation neighboring genes of EECN or NECN genes, I calculated the pairwise Spearman expression correlation coefficient, respectively. For each gene pair between EECN or NECN genes and their after-separation neighboring genes, I calculated the pairwise Spearman expression correlation coefficient, respectively. For each possible gene pair in *S. cerevisiae*, I also calculated the pairwise Spearman expression correlation coefficient. Distributions of resulting correlation coefficient values were presented for the five gene groups. To avoid confusion, I excluded gene pairs that were neighbors in any one species. The statistical significant values in **(A)** and **(B)** calculated from Mann–Whitney *U* test were indicated.

I examined whether after-separation neighboring genes of ECNS genes in S. cerevisiae are under selective constraints to maintain low-expression divergence of ECNS genes. I found that after-separation neighboring genes of ECNS genes show lower expression divergence than those of NECNS genes (P < 10–4, Mann–Whitney U test, **Figure 2A**). I asked whether ECNS genes show high coexpression with their after-separation neighboring genes to maintain their low-expression divergence. ECNS genes and their after-separation neighboring genes show higher coexpression than all possible gene pairs in S. cerevisiae (Thompson et al., 2013) (average coexpression level: 0.07 vs. 0.004, P < 10–6, Mann–Whitney U test, **Figure 2B**) but show similar coexpression levels compared with those between NECNS genes and their after-separation neighboring genes (average coexpression level: 0.07 vs. 0.04, P = 0.59, Mann–Whitney U test).

Considering the low-expression divergence of ECNS genes, if their evolutionary-conserved neighboring genes strongly influence their gene expression, after-separation gene neighbors that show high coexpression with their before-separation neighbors should be selectively favorable. Indeed, before- and after-separation neighboring genes of ECNS genes show higher coexpression than all possible gene pairs (average coexpression level: 0.04 vs. 0.004, *P* < 10^–11^, Mann–Whitney *U* test, **Figure 2B**). Before- and after-separation neighboring genes of ECNS genes also show higher coexpression than before- and after-separation neighboring genes of NECNS genes (average coexpression level: 0.04 vs. −0.14, *P* < 10^–20^, Mann–Whitney *U* test, **Figure 2B**). However, before- and after-separation neighboring genes of ECNS genes show lower coexpression than ECNS genes and their after-separation neighboring genes (*P* = 0.01, Mann–Whitney *U* test, **Figure 2B**). These results together implied that there is selective pressure for genes to insert in genomic neighborhoods that have similar expression noise (i.e., low-expression divergence), independent of whether genes are coexpressed. On the other hand, ECNS genes and their before-separation neighboring genes show comparable coexpression levels in *S. cerevisiae* with all possible gene pairs (average coexpression level: 0.01 vs. 0.004, *P* = 0.07, Mann–Whitney *U* test).

### Before- and After-Separation Neighboring Genes of ECNS Genes Tend to be in the Same Cellular Component

**Figure 3:**
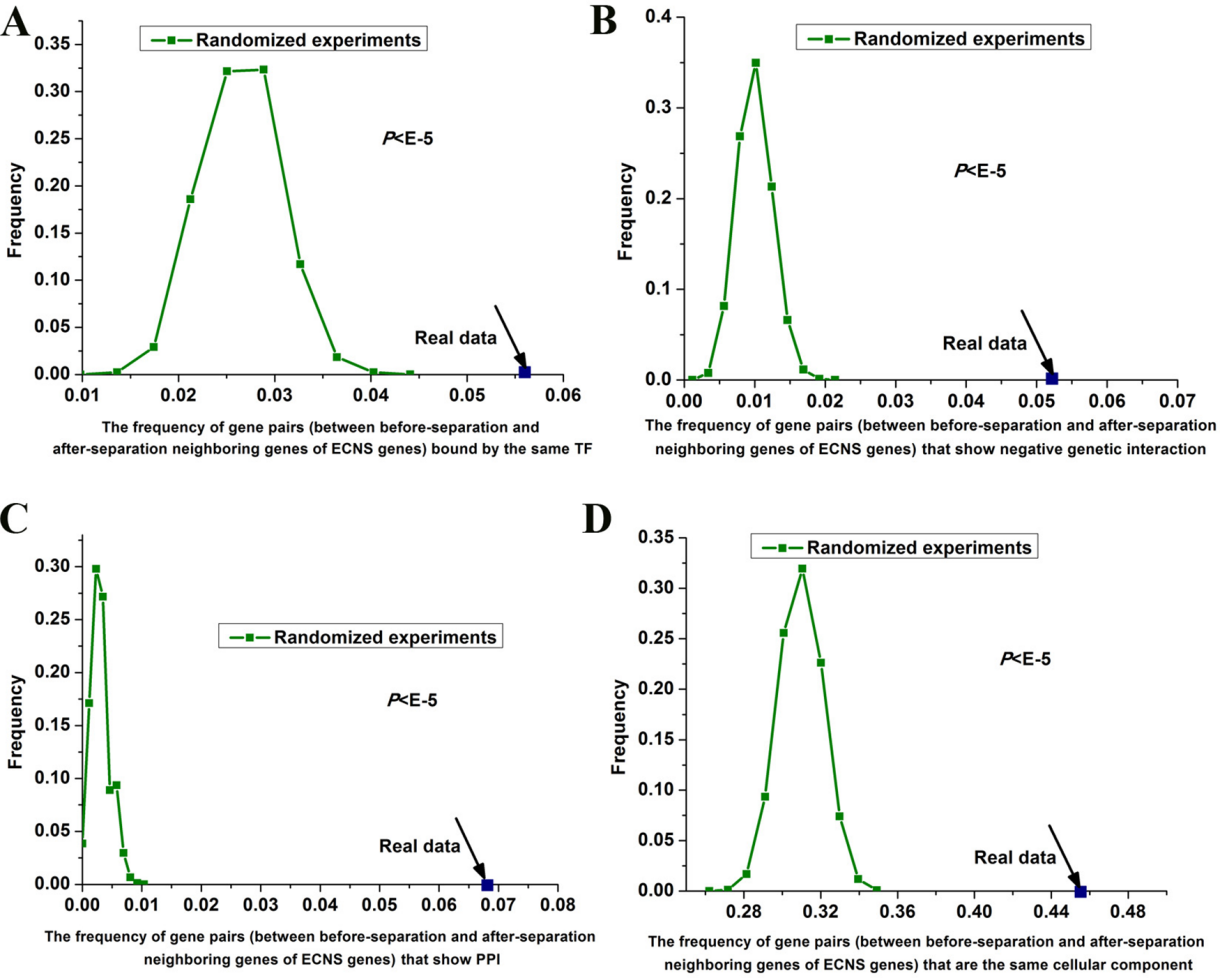
Gene pairs between before- and after-separation neighboring genes of ECNS genes tend to be in the same cellular component. Distributions of the frequencies of the gene pairs, between before- and after-separation neighboring genes of ECNS genes, which are bound by the same TF **(A)**, show negative pairwise genetic interactions **(B)**, show PPI **(C)**, in the same cellular component **(D)**. The dots were for the realistic data, while the lines depicted the distributions for 100,000 randomized experiments. The statistical significant values were indicated.

I tested whether before- and after-separation neighboring genes of ECNS genes are regulated by similar transcriptional programs. To address this, I used genome-wide 203 TF binding data in *S. cerevisiae* (Harbison et al., 2004). I generated random gene pairs to test the statistical significance (see details in Materials and methods section). I repeated the randomized experiment 100,000 times to calculate *P* value. I found that before- and after-separation neighboring genes of ECNS genes tend to be bound by the same TF: the frequencies of gene pairs bound by the same TF for all these randomized experiments were lower than that of the actual gene pairs (*P* < 10^–5^, **Figure 3A**).

Genetic interaction network plays an important role in the evolution of gene order (Yang et al., 2017). Using genome-wide genetic interaction network data in *S. cerevisiae* (Costanzo et al., 2016) and 100,000 times of randomized experiments, I found that gene pairs between before- and after-separation neighboring genes of ECNS genes tend to have pairwise genetic interactions (*P* < 10^–5^ for negative genetic interaction, **Figure 3B**, *P* = 0.02 for positive genetic interaction). Gene pairs between before- and after-separation neighboring genes of NECNS genes do not show these properties (*P* = 0.37 for negative genetic interaction, *P* = 0.64 for positive genetic interaction). As genes showing negative genetic interactions tend to encode proteins in the same protein complex (Costanzo et al., 2016), I tested whether it holds true for gene pairs between before- and after-separation neighboring genes of ECNS genes. Using genome-wide physical PPI data in *S. cerevisiae* (Stark et al., 2006) and 100,000 times of randomized experiments, I found that encoding proteins of gene pairs between before- and after-separation neighboring genes of ECNS genes tend to show pairwise PPI (P < 10^–5^, **Figure 3C**). Using Gene Ontology data in *S. cerevisiae* (Harris et al., 2004) and 100,000 times of randomized experiments, I found that gene pairs between before- and after-separation neighboring genes of ECNS genes tend to belong to the same cellular component (*P* < 10^–5^, **Figure 3D**).

## Discussion

Neighboring genes are frequently coexpressed. Whether this coexpression has consequent function or is just transcriptional noise remains debated (Xu et al., 2012; Kustatscher et al., 2017). If it is just the by-product of transcriptional noise, after genes separated from their evolutionary conserved neighboring genes, their after-separation gene neighbors should not be under selective constraints to show coexpression with their before-separation gene neighbors. Our results show that before- and after-separation neighboring genes of ECNS genes show statistical significantly higher coexpression than before- and after-separation neighboring genes of NECNS genes, although the mean coexpression level between before- and after-separation neighboring genes of ECNS genes is relatively low (0.04).

Using randomized experiments, I found that a statistically significant number of gene pairs between before- and after-separation neighboring genes of ECNS genes are bound by the same TF, to be in the same cellular component, show pairwise genetic interactions and PPI. These cofeatures are generally associated with gene coregulation. These results indicate that genes showing some similar specific properties with before-separation neighboring genes of ECNS genes tend to be selected as after-separation neighboring genes of ECNS genes. On the other hand, the numbers of gene pairs showing cofeatures differ among these properties. Significantly, ∼46% of gene pairs between before- and after-separation neighboring genes of ECNS genes are in the same cellular component (**Figure 3D**). It is likely that genes showing cocomponent with before-separation neighboring genes of ECNS genes are most favorable as after-separation neighboring genes of ECNS genes.

One interesting finding in this study is that ECNS genes and their after-separation neighbor genes show comparable coexpression levels with those between NECNS genes and their after-separation neighbor genes. I also identified nonseparation gene neighbors conserved from at least four yeast species to *S. cerevisiae*. These nonseparation gene neighbors show similar coexpression levels with those between ECNS genes and their after-separation neighbor genes (data not shown). The coexpression of neighboring genes is a phenomenon caused by many factors. The contribution of these factors varies with genes. Insights into mechanisms of regulation and gene order evolution from only neighboring coexpression information might be masked by some factors. Integrating neighboring coexpression information with gene neighborhood conservation information and before-separation neighboring information could provide deep insights into mechanisms of gene order evolution.

## Data Availability Statement

Publicly available datasets were analyzed in this study. This data can be found here: http://www.broad.mit.edu/regev/orthogroups/.

## Author Contributions

ZD designed the study, implemented the algorithms, carried out the experiments, analyzed the results, and drafted the manuscript.

## Funding

This work was supported by National Natural Science Foundation of China (NSFC) (grant nos. 61872395 and U1611265), by Natural Science Foundation of Guangdong Province (2018A030313285), and also by Pearl River Nova Program of Guangzhou (201710010044).

## Conflict of Interest

The author declares that the research was conducted in the absence of any commercial or financial relationships that could be construed as a potential conflict of interest.
