# Supplementary material for: Gene Repositioning Is Under Constraints After Evolutionary Conserved Gene Neighborhood Separate
Source: Front Genet. 2019 Oct 3;10:1030. doi: 10.3389/fgene.2019.01030 (PMC6785632; doi:10.3389/fgene.2019.01030)
Supplement: Supplementary file 1 [file DataSheet_1.pdf]

# *Supplementary Material*

## 1 Supplementary Figures

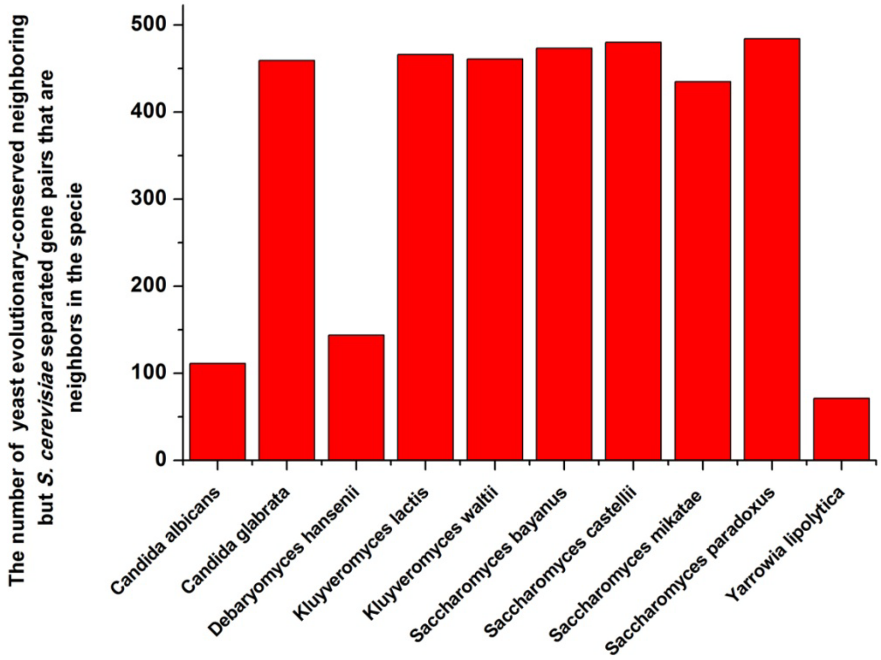


**Supplementary Figure 1.** For each species, the number of yeast evolutionary-conserved neighboring but *S. cerevisiae* separated gene pairs that are neighbors in this specie was indicated.

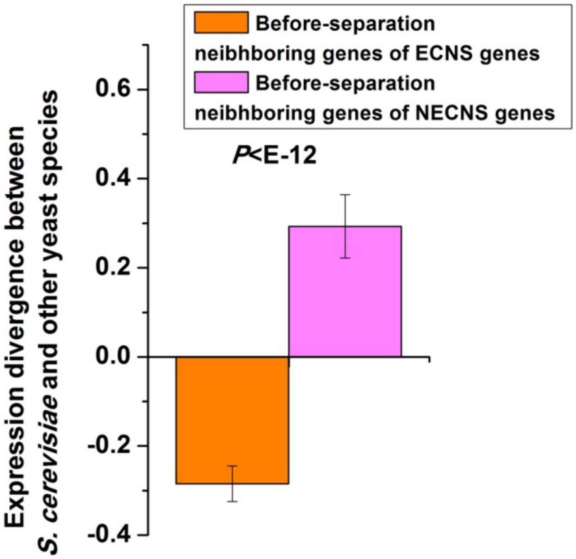


**Supplementary Figure 2**. Before-separation neighboring genes of ECNS genes also show lower expression divergence than those of NECNS genes. Median values that correspond to expression divergence between *S. cerevisiae* and other yeast species were shown for before-separation neighboring genes of ECNS genes and NECNS genes. Error bars were calculated by bootstrapping. The statistical significant value calculated from Mann-Whitney U-test was indicated.
